# Pregnancy in Women With Impaired Left Ventricular Function: Data From ROPAC

**DOI:** 10.1016/j.jacadv.2026.102605

**Published:** 2026-02-25

**Authors:** Puck N.J. Peters, Sahra Ünlütürk, Avraham Shotan, Lucia Baris, Laura Galian Gay, Niloufar Samiei, Mark R. Johnson, Helmut Baumgartner, Mette-Elise Estensen, Olga Irtyuga, Francesca M. Comoglio, Waltraut M. Merz, Roger Hall, Jolien W. Roos-Hesselink, ROPAC Investigators Group, Christopher Peter Gale, Branko Beleslin, Andrzej Budaj, Ovidiu Chioncel, Nikolaos Dagres, Nicolas Danchin, David Erlinge, Jonathan Emberson, Michael Glikson, Alastair Gray, Meral Kayikcioglu, Aldo Maggioni, Klaudia Vivien Nagy, Aleksandr Nedoshivin, Anna-Sonia Petronio, Jolien Roos-vHesselink, Lars Wallentin, Uwe Zeymer, Roger Hall, Jolien Roos-Hesselink, Joerg Stein, William Anthony Parsonage, Werner Budts, Julie De Backer, Jasmin Grewal, Ariane Marelli, Harald Kaemmerer, Guillaume Jondeau, Mark Johnson, Aldo P. Maggioni, Luigi Tavazzi, Ulf Thilen, Uri Elkayam, Catherine Otto, Karen Sliwa, A. Aquieri, A. Saad, H. Ruda Vega, J. Hojman, J.M. Caparros, M. Vazquez Blanco, M. Arstall, C.M. Chung, G. Mahadavan, E. Aldridge, M. Wittwer, Y.Y. Chow, W.A. Parsonage, K. Lust, N. Collins, G. Warner, R. Hatton, A. Gordon, E. Nyman, J. Stein, E. Donhauser, H. Gabriel, A. Bahshaliyev, F. Guliyev, I. Hasanova, T. Jahangirov, Z. Gasimov, A. Salim, C.M. Ahmed, F. Begum, M.H. Hoque, M. Mahmood, M.N. Islam, P.P. Haque, S.K. Banerjee, T. Parveen, M. Morissens, J. De Backer, L. Demulier, M. de Hosson, W. Budts, M. Beckx, M. Kozic, M. Lovric, T. Kovacevic-Preradovic, N. Chilingirova, P. Kratunkov, N. Wahab, S. McLean, E. Gordon, L. Walter, A. Marelli, A.R. Montesclaros, G. Monsalve, C. Rodriguez, F. Balthazar, V. Quintero, W. Palacio, L.A. Mejía Cadavid, E. Munoz Ortiz, F. Fortich Hoyos, E. Arevalo Guerrero, J. Gandara Ricardo, J. Velasquez Penagos, Z. Vavera, J. Popelova, N. Vejlstrup, L. Grønbeck, M. Johansen, A. Ersboll, Y. Elrakshy, K. Eltamawy, M. Gamal Abd-El Aziz, A. El Nagar, H. Ebaid, H. Abo Elenin, M. Saed, S. Farag, W. Makled, K. Sorour, Z. Ashour, G. El-Sayed, M. Abdel Meguid Mahdy, N. Taha, A. Dardeer, M. Shabaan, A. Saad, M. Ali, P. Moceri, G. Duthoit, M. Gouton, J. Nizard, L. Baris, S. Cohen, M. Ladouceur, L. Baris, D. Khimoud, B. Iung, F. Berger, A. Olsson, U. Gembruch, W.M. Merz, E. Reinert, S. Clade, Y. Kliesch, C. Wald, C. Sinning, R. Kozlik-Feldmann, S. Blankenberg, E. Zengin-Sahm, G. Mueller, M. Hillebrand, P. Hauck, Y. von Kodolitsch, N. Zarniko, H. Baumgartner, R. Schmidt, A. Hellige, O. Tutarel, H. Kaemmerer, B. Kuschel, N. Nagdyman, R. Motz, D. Maisuradze, A. Frogoudaki, E. Iliodromitis, M. Anastasiou-Nana, D. Triantafyllis, G. Bekiaris, H. Karvounis, G. Giannakoulas, D. Ntiloudi, S.A. Mouratoglou, A. Temesvari, H. Balint, D. Kohalmi, B. Merkely, C. Liptai, A. Nemes, T. Forster, A. Kalapos, K. Berek, K. Havasi, N. Ambrus, A. Shelke, R. Kawade, S. Patil, E. Martanto, T.M. Aprami, A. Purnomowati, C.J. Cool, M. Hasan, R. Akbar, S. Hidayat, T.I. Dewi, W. Permadi, D.A. Soedarsono, M.M. Ansari-Ramandi, N. Samiei, A. Tabib, F. Kashfi, S. Ansari-Ramandi, S. Rezaei, H. Ali Farhan, A. Al-Hussein, G. Al-Saedi, G. Mahmood, I.F. Yaseen, L. Al-Yousuf, M. AlBayati, S. Mahmood, S. Raheem, T. AlHaidari, Z. Dakhil, P. Thornton, J. Donnelly, M. Bowen, A. Blatt, A. Blatt, G. Elbaz-Greener, A. Shotan, S. Yalonetsky, S. Goland, M. Biener, G. Egidy Assenza, M. Bonvicini, A. Donti, A. Bulgarelli, D. Prandstraller, C. Romeo, R. Crepaz, E. Sciatti, M. Metra, R. Orabona, L. Ait Ali, P. Festa, V. Fesslova, C. Bonanomi, M. Calcagnino, F. Lombardi, A.M. Colli, M.W. Ossola, C. Gobbi, E. Gherbesi, L. Tondi, M. Schiavone, M. Squillace, M.G. Carmina, A. Maina, C. Macchi, E. Gollo, F.M. Comoglio, N. Montali, P. Re, R. Bordese, T. Todros, V. Donvito, W. Grosso Marra, G. Sinagra, B. D'Agata Mottolese, M. Bobbo, V. Gesuete, S. Rakar, F. Ramani, K. Niwa, D. Mekebekova, A. Mussagaliyeva, T. Lee, E. Mirrakhimov, S. Abilova, E. Bektasheva, K. Neronova, O. Lunegova, R. Žaliūnas, R. Jonkaitienė, J. Petrauskaitė, A. Laucevicius, D. Jancauskaite, L. Lauciuviene, L. Gumbiene, L. Lankutiene, S. Glaveckaite, M. Laukyte, S. Solovjova, V. Rudiene, K.H. Chee, C.C.-W. Yim, H.L. Ang, R. Kuppusamy, T. Watson, M. Caruana, M.-E. Estensen, M.G.A. Mahmood Kayani, R. Munir, A. Tomaszuk-Kazberuk, B. Sobkowicz, J. Przepiesc, A. Lesniak-Sobelga, L. Tomkiewicz-Pajak, M. Komar, M. Olszowska, P. Podolec, S. Wisniowska-Smialek, M. Lelonek, U. Faflik, A. Cichocka-Radwan, K. Plaskota, O. Trojnarska, N. Guerra, L. de Sousa, C. Cruz, V. Ribeiro, S. Jovanova, V. Petrescu, R. Jurcut, C. Ginghina, I. Mircea Coman, M. Musteata, O. Osipova, T. Golivets, I. Khamnagadaev, O. Golovchenko, A. Nagibina, I. Ropatko, I.R. Gaisin, L. Valeryevna Shilina, N. Sharashkina, E. Shlyakhto, O. Irtyuga, O. Moiseeva, E. Karelkina, I. Zazerskaya, A. Kozlenok, I. Sukhova, L. Jovovic, K. Prokšelj, M. Koželj, A.O. Askar, A.A. Abdilaahi, M.H. Mohamed, A.M. Dirir, K. Sliwa, P. Manga, A. Pijuan-Domenech, L. Galian-Gay, P. Tornos, M.T. Subirana, M.T. Subirana, N. Murga, J.M. Oliver, B. Garcia-Aranda Dominguez, I. Hernandez Gonzalez, J.F. Delgado Jimenez, P. Escribano Subias, A. Elbushi, A. Suliman, K. Jazzar, M. Murtada, N. Ahamed, M. Dellborg, E. Furenas, M. Jinesjo, K. Skoglund, P. Eriksson, T. Gilljam, U. Thilen, D. Tobler, K. Wustmann, F. Schwitz, M. Schwerzmann, T. Rutz, J. Bouchardy, M. Greutmann, B.M. Santos Lopes, L. Meier, M. Arrigo, K. de Boer, T. Konings, E. Wajon, L.J. Wagenaar, P. Polak, E.P.G. Pieper, J. Roos-Hesselink, L. Baris, I. van Hagen, H. Duvekot, J.M.J. Cornette, C. De Groot, C. van Oppen, L. Sarac, O. Batukan Esen, S. Catirli Enar, C. Mondo, P. Ingabire, B. Nalwanga, T. Semu, B.T. Salih, W.A.R. Almahmeed, S. Wani, F.S. Mohamed Farook, F. Gerges, A.M. Komaranchath, F. Al bakshi, A. Al Mulla, A.H. Yusufali, E.I. Al Hatou, N. Bazargani, F. Hussain, L. Hudsmith, P. Thompson, S. Thorne, S. Bowater, A. Money-Kyrle, P. Clifford, P. Ramrakha, S. Firoozan, J. Chaplin, N. Bowers, D. Adamson, F. Schroeder, R. Wendler, S. Hammond, P. Nihoyannopoulos, R. Hall, L. Freeman, G. Veldtman, J. Kerr, L. Tellett, N. Scott, A.B. Bhatt, D. DeFaria Yeh, M.A. Youniss, M. Wood, A.A. Sarma, S. Tsiaras, A. Stefanescu, J.M. Duran, L. Stone, D.S. Majdalany, J. Chapa, K. Chintala, P. Gupta, J. Botti, J. Ting, W.R. Davidson, G. Wells, D. Sparks, V. Paruchuri, K. Marzo, D. Patel, W. Wagner, S.N. Ahanya, L. Colicchia, T. Jentink, K. Han, M. Loichinger, M. Parker, W. Wagner, C. Longtin, A. Yetman, K. Erickson, J. Cramer, S. Tsai, B. Fletcher, S. Warta, C. Cohen, C. Lindblade, R. Puntel, K. Nagaran, N. Croft, M. Gurvitz, C. Otto, C. Talluto, D. Murphy, M.G. Perlroth

**Affiliations:** a Department of Cardiology, Erasmus Medical Center, Rotterdam, the Netherlands; b Department of Cardiology, Hillel Yaffe Medical Center, Hadera, Israel; c Department of Cardiology, University Hospital Vall d’Hebron, Barcelona, Spain; d CIBERCV, Instituto de Salud Carlos III, Madrid, Spain; e Rajaei Cardiovascular Medical and Research Center, Tehran, Iran; f Department of Obstetric Medicine, Imperial College London, London, United Kingdom; g Department of Cardiology, University Hospital Muenster, Muenster, Germany; h Department of Cardiology, Oslo University Hospital, Oslo, Norway; i Department of Cardiology, Almazov Federal Medical Research Centre, Saint Petersburg, Russia; j Department of Surgical Sciences, University of Torino, Torino, Italy; k Department of Obstetric Medicine, University Hospital of Bonn, Bonn, Germany; l Department of Cardiology, University of East Anglia, Norwich, United Kingdom

**Keywords:** cardiomyopathy, heart disease, heart failure, maternal mortality, pregnancy

## Abstract

**Background:**

The hemodynamic changes during pregnancy can be challenging in women with underlying heart disease, particularly in women with impaired left ventricular function (LVF, left ventricular ejection fraction <40%).

**Objectives:**

The aim of this study was to describe the cardiac, obstetric, and fetal outcomes of pregnancy in women with impaired LVF.

**Methods:**

ROPAC (Registry Of Pregnancy and Cardiac disease) includes an international, prospective, observational cohort of pregnancies in women with heart disease. Cardiac, obstetric, and fetal outcomes were analyzed in 251 patients with impaired LVF. The primary endpoint was the occurrence of major adverse cardiac events (MACE) including maternal death, supraventricular or ventricular arrhythmias, heart failure, aortic dissection, endocarditis, ischemic coronary event, and other thromboembolic events. Logistic regression was used to determine variables associated with poor outcomes.

**Results:**

Median follow-up duration was 7 (6-11) months. Maternal mortality occurred in 6/251 (2.4%, 1%-5%) and heart failure in 67/251 (27%, 21%-33%) patients. Ventricular tachyarrhythmias occurred in 11/251 (4%, 2%-8%) patients. Eighty-one of 251 (32%, 27%-38%) patients experienced at least one MACE during pregnancy or up to 6 months postpartum. Obstetric complications were common, including preterm birth in 67/251 (27%, 22%-33%) and low birthweight in 65/251 (26%, 21%-32%). Patients with cardiomyopathy were at higher risk of cardiovascular complications with 4.3% mortality and nearly 40% risk of MACE during pregnancy. Prepregnancy signs of heart failure (OR: 2.67; 1.3-5.6), atrial fibrillation (OR: 6.32; 3.0-13.3), and an NYHA functional class >II (OR: 6.06; 2.2-16.6) were associated with poor cardiac outcomes.

**Conclusions:**

Women with impaired LVF are at increased risk of complications, particularly heart failure, tachyarrhythmias, and premature delivery with low birth weight.

Impaired left ventricular function (LVF) predominantly affects the elderly, with only 0.2 percent of young adults being affected.^1^ This figure is rising due to the increasing frequency of premature atherosclerosis, the improved life expectancy of patients with congenital heart disease (CHD), the better standard of care for cardiomyopathy (CMP) patients, and the increasing incidence of chemotherapy-induced LV dysfunction.^2^

During pregnancy, a variety of hemodynamic changes occur which are necessary for the mother to be able to support the growing fetus.^3^, ^4^, ^5^ These changes can be challenging for women with underlying maternal heart disease, particularly with impaired LVF when the physiological increase in cardiac output and heart rate during pregnancy can have devastating consequences.

According to the modified World Health Organization (mWHO) classification, pregnant women with mild LV impairment are in mWHO class II-III and therefore have a small to significantly increased risk of maternal mortality and severe morbidity. However, the presence of severe LV impairment with an LV ejection fraction (LVEF) < 30% is considered a contraindication for pregnancy (mWHO class IV).^6^ The cardiac disease in pregnancy (CARPREG) risk score is also widely used and suggests that the presence of systemic ventricular dysfunction, with an LVEF <40%, is an independent predictor of cardiac complications during pregnancy.^7^ However, due to the low incidence of impaired LVF in pregnancy, most of these recommendations are based on expert opinion, small case series, which are mostly retrospective, and on women with a variety of lesions. Indeed, CARPREG includes patients with complex (corrected) congenital malformations with a systemic right ventricle or a Fontan circulation, emphasizing that there is a lack of specific information for patients with impaired LVF.

The current prospective study aims to provide information on the cardiac, obstetric, and fetal outcomes in a cohort of pregnant women with impaired LVF (LVEF <40%), excluding patients with complex CHD, derived from the worldwide observational European Society of Cardiology (ESC) EURObservational Research Programme (EORP) Registry Of Pregnancy And Cardiac disease (ROPAC), and to identify associations with adverse pregnancy outcomes.

## Methods

### Study design

The ESC-EORP ROPAC is an international, prospective, observational registry of pregnant patients with structural or ischemic heart disease, aorta pathology, and pulmonary arterial hypertension. The study design and methods have been described in detail previously.^8^ When required, ethical approval or Institutional Review Board approval was obtained (eg, in Germany, United States, Canada, and Belgium). However, in some countries, the procedure to obtain ethical approval was waived because of the anonymized and untraceable nature of the data. Informed consent was obtained from patients if required by the local independent review board. Pregnant patients were included prospectively from 2008, and for this analysis, we included all pregnancies in patients with impaired LVF enrolled between January 2008 and January 2018. We compared outcome of our patients' series with ROPAC patients with normal LVF (Central Illustration). Patients with a systemic right ventricle or a univentricular heart were excluded.

**Central Illustration:**
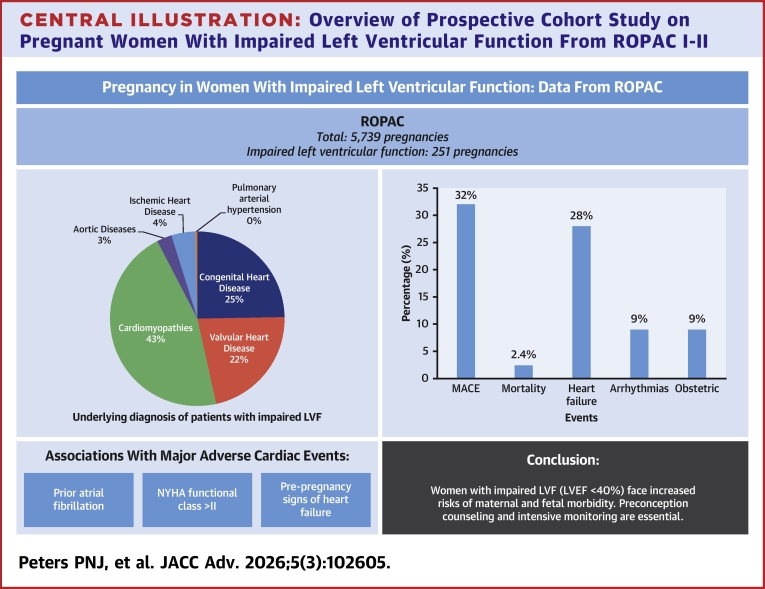
Overview of Prospective Cohort Study on Pregnant Women With Impaired Left Ventricular Function From ROPAC I-II MACE = major adverse cardiac events; ROPAC = Registry of Pregnancy and Cardiac disease.

### Data

The ROPAC study protocol and the first results of this registry were published in 2013.^8^ Baseline characteristics collected before pregnancy included age, NYHA functional classification, electrocardiogram rhythm, diagnosis, risk factors (smoking habits, hypertension, diabetes, signs of heart failure [in the past or pre-existing]), medication, previous interventions, parity and obstetric history and echocardiographic measurements. Countries were divided into developed or emerging countries according to the International Monetary Fund Classification. Data were available up to 1 week postpartum for all patients; data on the 6-month postpartum follow-up were available in 182 patients (73%).

### Definitions and endpoints

The primary combined endpoint was the occurrence of a major adverse cardiac event (MACE), defined as combined endpoint of maternal death, supraventricular or ventricular arrhythmias requiring treatment, heart failure, aortic dissection, endocarditis, ischemic coronary event, and other thromboembolic events. The secondary endpoints were adverse obstetric outcomes and adverse fetal/neonatal outcomes. Heart failure was defined according to the American College of Cardiology/American Heart Association guidelines, and heart failure episodes were only included when they required hospital admission, new treatment, or a change in the existing treatment regimen. Impaired LVF was defined as an LVEF <40%. Postpartum hemorrhage was defined as increased blood loss during delivery up to 24 hours postpartum requiring specific interventions. Hemolysis, elevated liver enzymes, low platelets syndrome, eclampsia and pre-eclampsia, and pregnancy-induced hypertension were defined according to the International Society for the Study of Hypertension in Pregnancy 2012 statement.^9^ Fetal mortality was defined as the death of a fetus after 20 weeks gestational age until birth. Neonatal mortality was defined as the death of a live-born baby in the first 6 months of life. Premature birth was defined as birth before 37 weeks gestational age. Low birth weight was defined as a birth weight below 2,500 g. Low Apgar score was defined as an Apgar score at 5 minutes of below 7. All outcomes were examined for the duration of the pregnancy and up to 6 months postpartum.

### Statistical analysis

Data are presented as mean ± SD if normally distributed and median (IQR) if skewed. Categorical data are presented as frequencies and percentages. Differences between groups were assessed using Student’s *t*-test for normally distributed variables and the Mann-Whitney *U* test for skewed distributed variables. Differences in categorical variables were assessed using the chi-square test or Fisher exact test (>20% of expected cell counts <5), if appropriate. Univariable analyses were performed to explore associations between variables and MACE, and not causal relationships. Variables showing a significant association (*P* ≤ 0.05) in univariable analyses were subsequently entered into a multivariable logistic regression model with a logit link to explore associations with MACE. Missing data in prespecified variables (age, nulliparity, NYHA functional class >II, prior medication, anticoagulation, history of hypertension, and signs of heart failure) were handled using multiple imputation by chained equations (Supplemental Tables 1 and 2). Using multiple imputation by chained equations, 5 data sets were generated using 5 iterations. Subsequently, regression models were all fitted on each data set separately, and model coefficients and *P* values were pooled according to Rubin’s rules. Supplemental Tables 1 and 2 summarize the proportion of missing data for each variable included in the multivariable analyses. Complete-case analyses yielded similar results and are provided for sensitivity. All statistical tests and analyses were performed with SPSS version 21.0 (SPSS Inc). A *P* value of ≤0.05 (2-sided test) was considered significant.

## Results

**Figure 1:**
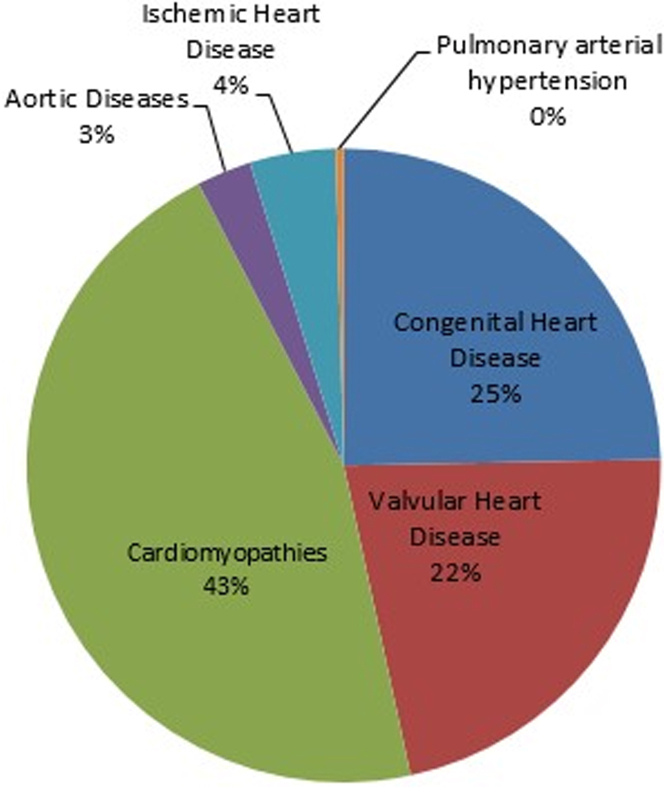
Diagnosis Details of 251 Pregnant Women With Impaired Left Ventricular Function

Of the 5,739 pregnancies within ROPAC, a total of 251 pregnancies in patients with impaired LVF were included from 50 centers in 32 countries, of which 16 were countries with an advanced economy. Four out of 251 (2%) pregnancies were twin pregnancies. Median follow-up duration was 7 (6-11) months. The mean maternal age was 30.8 ± 5.6 years, and 98/251 (39%) patients were nulliparous. Baseline characteristics are presented in Table 1. Differences between the patients with and without impaired LVF were found in the distribution of underlying diagnoses and in the baseline parameters. The underlying diagnoses of patients with impaired LVF are shown in Figure 1. One hundred fifteen out of 251 (46%) patients had some form of CMP, and 64/251 (26%) patients presented with signs of heart failure prior to pregnancy. The majority of patients were in functional NYHA functional class I (122/251, 49%). Prior to pregnancy, 169/251 (67%) patients were treated with cardiac medication, of which 87 (35%) were taking a beta-blocker, 9 (4%) diuretics, and 21 (8%) an angiotensin-converting enzyme-inhibitor (ACE-inhibitor) (Supplemental Table 3).

**Table 1:** Baseline Characteristics

	Impaired LVF (n = 251)	ROPAC Cohort (Normal LVF) (n = 5,369)	*P* Value
Demographics			
Age in y	30.8 ± 5.6	29.5 ± 5.7	0.001
Nulliparity	98 (39%)	2,406 (45%)	0.040
Emerging country	136 (54%)	2,129 (40%)	<0.001
Diagnosis			
Cardiomyopathy	115 (46%)	323 (6%)	<0.001
Dilated cardiomyopathy	53 (46.1%^a^)	31 (9.6%^a^)	<0.001
Hypertrophic cardiomyopathy	5 (4.3%^a^)	88 (27.2%^a^)	0.630
Other CMP^a^	57 (49.6%^a^)	111 (34.4%^a^)	<0.001
Congenital heart disease	62 (25%)	3,114 (58%)	<0.001
Tetralogy of Fallot	11 (18%^b^)	415 (13%^b^)	0.060
Transposition of the great arteries (arterial switch)	2 (3%^b^)	178 (6%^b^)	0.030
Atrial or ventricular septal defects	17 (27%^b^)	1,111 (36%^b^)	<0.001
Pulmonary atresia	2 (3%^b^)	34 (1%^b^)	0.730
Valvular heart disease	55 (22%)	1,593 (30%)	0.010
Aortic stenosis	1 (2%^c^)	137 (9%^c^)	0.030
Aortic regurgitation	2 (4%^c^)	146 (9%^c^)	0.070
Aortic stenosis and regurgitation	1 (2%^c^)	69 (4%^c^)	0.230
Mitral stenosis	15 (27%^c^)	273 (17%^c^)	0.480
Mitral regurgitation	18 (33%^c^)	482 (30%^c^)	0.380
Mitral stenosis and regurgitation	15 (27%^c^)	263 (17%^c^)	0.390
Pulmonary stenosis	2 (4%^c^)	100 (63%^c^)	0.230
Ischemic heart disease	11 (4%)	84 (2%)	0.001
Aortic pathology	7 (3%)	210 (4%)	0.370
Pulmonary arterial hypertension	1 (0.4%)	44 (1%)	0.460
Prepregnancy history			
Current smoking	15 (6%)	209 (4%)	0.110
Hypertension	33 (13%)	345 (6%)	<0.001
Heart failure	64 (26%)	525 (10%)	<0.001
Atrial fibrillation	11 (4%)	95 (2%)	0.003
Angina pectoris	14 (6%)	155 (3%)	0.040
Diabetes mellitus	4 (2%)	85 (2%)	0.120
NYHA functional class I	122 (49%)	4,007 (75%)	<0.001
NYHA functional class II	85 (34%)	1,074 (20%)	<0.001
NYHA functional class III	28 (11%)	145 (3%)	<0.001
NYHA functional class IV	6 (2%)	21 (0.4%)	<0.001
Prior intervention	96 (38%)	2,974 (55%)	<0.001
Prior medication	169 (67%)	1,552 (29%)	<0.001
Beta-blocker	87 (35%)	350 (7%)	<0.001
Diuretics	9 (4%)	39 (0.7%)	<0.001
ACE inhibitor	21 (8%)	52 (1%)	<0.001
Anticoagulation	96 (38%)	1,018 (19%)	<0.001

Values are mean ± SD or n (%).

ACE = angiotensin-converting enzyme; CHD = congenital heart disease; CMP = cardiomyopathy; LVF = left ventricular function; ROPAC = Registry of Pregnancy and Cardiac disease; VHD = valvular heart disease.

a Percentage of subdiagnosis CMP.

b Percentage of subdiagnosis CHD.

c Percentage of subdiagnosis VHD.

### Maternal cardiac outcome

In 81/251 (32%; 95% CI: 27%-38%) patients, at least one MACE occurred during pregnancy or postpartum, of which 63 (25%; 95% CI: 20%-31%) patients required hospital admission. Pregnancy was complicated by heart failure in 67/251 (27%; 95% CI: 21%-33%) and 12/251 (5%; 95% CI: 3%-9%) women developed heart failure postpartum, of which 2 (1%) developed heart failure for the first time after delivery (10 weeks and 5 months postpartum) and 1 (0.3%) developed heart failure during pregnancy secondary to mechanical valve thrombosis. Arrhythmias during pregnancy occurred in 22/251 (9%; 95% CI: 6%-13%) patients, with atrial fibrillation or flutter in 11/251 (4%; 95% CI: 3%-8%) and ventricular tachyarrhythmias in 11/251 (4%; 95% CI: 2%-8%) patients. The cardiovascular (CV) outcomes of patients with impaired LVF are summarized and compared to heart disease patients with normal LVF in Table 2. Almost all patients who were in NYHA functional classes III (79%) or IV (83%) prior to pregnancy experienced a cardiac event during pregnancy or in the postpartum period, as presented in Supplemental Figure 1. The highest rate of MACE during pregnancy was seen in patients with dilated CMP (24/53, 45%), hypertrophic CMP (1/5, 20%), combined valvular disease of mitral stenosis and mitral regurgitation (11/16, 67%), and in patients with peripartum CMP in previous pregnancies (12/25, 48%).

**Table 2:** Cardiovascular Outcome

	Impaired LVF (n = 251)	ROPAC Cohort (Normal LVF) (n = 5,369)	*P* Value
MACE	81 (32%, 27%-38%)	698 (13%, 12%-14%)	<0.001
Maternal mortality	6 (2.4%, 1%-5%)	33 (0.6%, 0.4%-1%)	0.001
Hospital admission for cardiac reasons	63 (25%, 20%-31%)	673 (13%, 12%-14%)	<0.001
Heart failure episode during pregnancy	67 (27%, 21%-33%)	532 (10%, 9%-11%)	<0.001
Heart failure episode postpartum	12 (5%, 3%-9%)	78 (1.5%, 1.2%-2%)	<0.001
Ventricular tachyarrhythmia	11 (4%, 2%-8%)	75 (1.4%, 1.1%-1.7%)	<0.001
Atrial fibrillation or flutter	11 (4%, 3%-8%)	78 (1.5%, 1.2%-1.8%)	<0.001
Endocarditis	1 (0.4%, 0.1%-2%)	31 (0.6%, 0.4%-0.8%)	0.710
Aortic dissection	0 (0%)	5 (0.1%, 0.04%-0.2%)	0.630
Ischemic coronary event	2 (0.8%, 0.2%-3%)	22 (0.4%, 0.3%-0.6%)	0.360
Other thromboembolic events^a^	8 (3.2%, 1%-5%)	74 (1.4%, 1.1%-1.7%)	0.020

Values n (%, 95% CI).

MACE = major adverse cardiac events; other abbreviations as in Table 1.

a Cerebrovascular accident or deep vein thrombosis.

### Maternal mortality

Six out of 251 (2.4%) patients died during pregnancy or in the postpartum period. Their age, underlying diagnosis, LVEF prior to pregnancy, NYHA functional class prior to pregnancy, cause of death, and timing of death have been summarized in Table 3.

**Table 3:** Details of Patients Who Died During Pregnancy or in the Postpartum Period

	Age, y	Diagnosis	LVEF	NYHA Functional Class	Cause of Death	Timing of Death
Patient 1	29	Dilated CMP	Unknown	II	Unknown	14 weeks GA
Patient 2	22	Dilated CMP	Unknown	II	Septic shock after heart transplant	14 weeks postpartum
Patient 3	35	Mechanical valve	30%	II	Refractory cardiogenic shock after developing severe heart failure at 20 weeks GA	1 week postpartum
Patient 4	24	Dilated CMP	25%	IV	Cardiogenic shock after developing pulmonary edema at 37 weeks GA	Two weeks postpartum
Patient 5	22	Noncompaction CMP	19%	IV	Refractory cardiogenic shock after developing heart failure and severe anemia	Unknown number of weeks after pregnancy termination in first trimester
Patient 6	30	Hypertrophic CMP	Unknown	III	Multi-organ failure due to refractory cardiogenic shock	Shortly after miscarriage at 22 weeks GA

GA = gestational age; LVEF = left ventricular ejection fraction; other abbreviation as in Table 1.

### Cardiac medication before and during pregnancy

A total of 169/251 (67%) patients were treated with cardiac medication before pregnancy. Of the 21/251 (8%) patients who used an ACE-inhibitor before pregnancy, 8 (38%) of them continued during pregnancy. The medications used before and during pregnancy are presented in Supplemental Table 3. The women who were using ACE inhibitors during pregnancy did not have significantly more children with congenital diseases or birth defects than women who did not use ACE-inhibitors during pregnancy (11.1% vs 2.9%; *P* = 0.170).

### Anticoagulation

Prior to pregnancy, 27/251 (11%) patients used vitamin K antagonists (VKAs). These were primarily patients with valvular heart disease (VHD) (n = 11, 63%) and with CHD (n = 4, 15%). Of the 27 patients with prepregnancy VKA use, 12 (44%) patients had one or more mechanical valvular prostheses (all left-sided) in situ. Of the 27 VKA users, 20 (74%) patients switched to low-molecular-weight heparin in therapeutic dosage during the first trimester. In the second and third trimester, 25 (93%) patients used VKA as anticoagulants, with a switch to either therapeutic or prophylactic dosages of low-molecular-weight heparin in the weeks before and during delivery (all except one). Additional 20/251 (8%) patients were using antiplatelet therapy (acetylsalicylic acid), which they continued throughout the pregnancy. In 8/251 (3%) patients, antiplatelet therapy was initiated during pregnancy.

### Associations with adverse cardiac outcome

**Figure 2:**
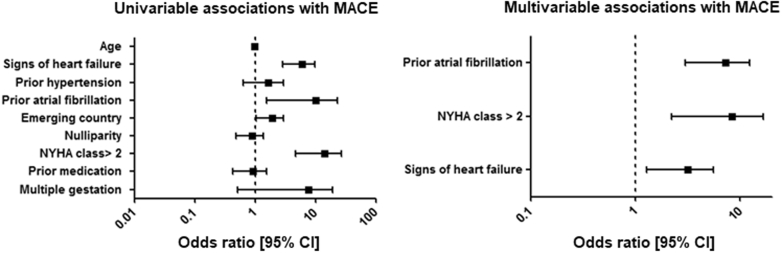
Associations With MACE in Women With Impaired LVF MACE = major adverse cardiac event; other abbreviation as in Figure 1.

The results of the univariable and multivariable logistic regressions are shown in Figure 2 and Supplemental Table 4. Prepregnancy signs of heart failure (OR: 2.67; 95% CI: 1.3-5.6), NYHA functional class >II (OR: 6.06; 95% CI: 2.2-16.6), and prior atrial fibrillation (OR: 6.32; 95% CI: 3.0-13.3) were associated with MACE, as observed in exploratory multivariable analyses.

### Subgroup analyses

#### Cardiomyopathy

Of the 115/251 (46%) CMP patients with impaired LVF, almost half (n = 55, 48%) had dilated CMP. Other frequently observed diagnoses were peripartum CMP (n = 25, 22%), chemotherapy-induced CMP (n = 7, 6%), LV noncompaction (n = 12, 10%), and myocarditis (n = 6, 5%). Baseline characteristics of CMP patients with impaired LVF are shown in Supplemental Table 5 and are compared to CMP patients with normal LVF. There were several differences found in underlying diagnoses and baseline parameters. Supplemental Table 6 depicts the CV outcome of CMP patients with impaired LVF in comparison to CMP patients with normal LVF. Maternal mortality rates (4.3% vs 0.9%; *P* = 0.020) and noncoronary thromboembolic complications (3.5% vs 0%) differed significantly. Table 4 shows the characteristics of patients with impaired LVF per diagnosis group, comparing patients with and without a CV event during pregnancy or postpartum. In the CMP group, a greater proportion with a CV event came from an emerging country (69% vs 50%; *P* = 0.046), showed signs of heart failure prepregnancy (49% vs 14%; *P* < 0.001), were in NYHA functional class III (20% vs 1%; *P* = 0.001) and NYHA functional class IV (11% vs 1%; *P* = 0.020) and a lesser proportion were in NYHA functional class I (18% vs 49%; *P* = 0.001).

**Table 4:** Characteristics of Patients With Impaired LVF per Diagnosis Group, Compared Between Patients With and Without a CV Event

	Cardiomyopathy			Congenital Heart Disease			Valvular Heart Disease		
	MACE (n = 45)	No MACE (n = 70)	*P* Value	MACE (n = 13)	No MACE (n = 49)	*P* Value	MACE (n = 21)	No MACE (n = 34)	*P* Value
Demographics									
Age in y	30.9 ± 6.7	31.8 ± 4.7	0.660	28.6 ± 6.9	28.8 ± 5.1	0.900	30.3 ± 6.4	29.6 ± 5.7	0.070
Nulliparity	15 (33%)	25 (36%)	0.790	7 (54%)	29 (59%)	0.730	4 (19%)	12 (35%)	0.200
Emerging country	31 (69%)	35 (50%)	0.046	4 (31%)	16 (33%)	0.890	16 (76%)	24 (71%)	0.650
Prepregnancy history									
Current smoking	3 (7%)	6 (9%)	0.030	0 (0%)	2 (4%)	0.070	1 (5%)	1 (3%)	0.260
Hypertension	10 (22%)	10 (14%)	0.270	1 (8%)	3 (6%)	0.860	2 (10%)	5 (15%)	0.820
Heart failure	22 (49%)	10 (14%)	<0.001	6 (46%)	4 (8%)	0.001	10 (48%)	9 (26%)	0.090
Atrial fibrillation	0 (0%)	0 (0%)	-	1 (8%)	0 (0%)	0.050	7 (33%)	3 (9%)	0.020
Angina pectoris	2 (4%)	4 (6%)	0.910	1 (8%)	1 (2%)	0.520	0 (0%)	3 (9%)	0.180
Diabetes mellitus	1 (2%)	0 (0%)	0.210	0 (0%)	0 (0%)	-	0 (0%)	0 (0%)	-
NYHA functional class I	8 (18%)	34 (49%)	0.001	5 (38%)	38 (78%)	0.007	4 (19%)	20 (59%)	0.004
NYHA functional class II	22 (49%)	29 (41%)	0.430	4 (31%)	8 (16%)	0.240	8 (38%)	9 (26%)	0.370
NYHA functional class III	9 (20%)	1 (1%)	0.001	3 (23%)	2 (4%)	0.030	9 (43%)	3 (9%)	0.003
NYHA functional class IV	5 (11%)	1 (1%)	0.020	0 (0%)	0 (0%)	-	0 (0%)	0 (0%)	-
Prior intervention	3 (7%)	8 (11%)	0.390	10 (77%)	37 (76%)	0.920	10 (48%)	19 (56%)	0.380
Prior medication	36 (80%)	55 (79%)	0.850	9 (69%)	13 (27%)	0.004	20 (95%)	22 (65%)	0.010
Beta-blocker	18 (40%)	38 (54%)	0.140	4 (31%)	6 (12%)	0.110	4 (19%)	8 (24%)	0.700
Diuretics	2 (4%)	4 (6%)	0.770	0 (0%)	1 (2%)	0.600	1 (5%)	0 (0%)	0.200
ACE inhibitor	6 (13%)	10 (14%)	0.890	0 (0%)	1 (2%)	0.600	1 (5%)	2 (6%)	0.860
Anticoagulation	17 (38%)	24 (34%)	0.700	4 (31%)	9 (18%)	0.330	16 (76%)	16 (47%)	0.030
Mechanical prosthesis	-	-	-	-	-	-	6 (29%)	9 (26%)	0.870

Values are mean ± SD or n (%).

CV = cardiovascular; other abbreviations as in Tables 1 and 2.

#### Congenital heart disease

Atrial and ventricular septal defects were the most frequently observed diagnoses in the CHD group with impaired LVF (17/62, 27%). Baseline characteristics of CHD patients with impaired LVF are shown in Supplemental Table 7 and are compared to CHD patients with normal LVF. Signs of heart failure prepregnancy were more often seen in the patients with impaired LVF (16% vs 6%; *P* = 0.001), as well as beta-blocker use prior to pregnancy (16% vs 5%; *P* < 0.001). Supplemental Table 8 shows the CV outcomes of CHD patients with impaired LVF in comparison to CHD patients with normal LVF. MACE rates (21% vs 8%; *P* < 0.001), especially heart failure episodes (19% vs 6%; *P* < 0.001) differed significantly. In the CHD group with a CV event, a significantly higher percentage of patients showed signs of heart failure prepregnancy (46% vs 8%; *P* = 0.001), had atrial fibrillation prepregnancy (8% vs 0%; *P* = 0.050), had received cardiac medications more often prior to pregnancy (69% vs 27%; *P* = 0.004), and were more often in NYHA functional class III (23% vs 4%; *P* = 0.030) and less often in NYHA functional class I (38% vs 78%; *P* = 0.007) (Table 3).

#### Valvular heart disease

Baseline characteristics of VHD patients with impaired LVF are shown in Supplemental Table 10 and are compared to VHD patients with normal LVF. Those with impaired LVF were more likely to have mitral stenosis alone or in combination with regurgitation (*P* < 0.05 and 0.04 respectively) and to have a mechanical valve (27% vs 17%; *P* = 0.040); they were more likely to have a pre-existing diagnosis of hypertension (13% vs 4%; *P* = 0.003), atrial fibrillation (18% vs 5%; *P* < 0.001) and heart failure (35% vs 16%; *P* < 0.001) and to be on medication; beta-blockers (22% vs 6%; *P* < 0.001), anticoagulation (58% vs 29%; *P* < 0.001), and ACE inhibitors (6% vs 1%; *P* = 0.002). Supplemental Table 10 shows the CV outcome of VHD patients with impaired LVF in comparison to VHD patients with normal LVF. MACE rates (38% vs 20%, *P* < 0.001), especially heart failure episodes during pregnancy (31% vs 16%; *P* = 0.002) and atrial fibrillation or flutter (13% vs 3%; *P* < 0.001) were more common. In the VHD with a CV event group, more patients suffered from atrial fibrillation prior to pregnancy (33% vs 9%; *P* = 0.020) and used prepregnancy medications (95% vs 65%; *P* = 0.010), in particular anticoagulation (76% vs 47%; *P* = 0.030); they were more often in NYHA functional class III (43% vs 9%; *P* = 0.003) and less often in NYHA functional class I (19% vs 59%; *P* = 0.004) (Table 4).

#### Obstetric and neonatal outcome

The obstetric and neonatal outcomes of patients with impaired LVF are summarized and compared to heart disease patients with normal LVF in Table 5. A total of 62/251 (25%; 95% CI: 20%-31%) pregnancies were complicated by one or more adverse obstetric events, and fetal events occurred in 80/251 (32%; 95% CI: 27%-38%) pregnancies. Patients with impaired LVF were more often delivered by Cesarean section (CS) (56% vs 43%; *P* < 0.001), which was more often an emergency (18% vs 9%; *P* < 0.001) and for cardiac reasons (6% vs 1%; *P* < 0.001). Despite experiencing more preterm birth (27% vs 15%; *P* < 0.001), low birth weight (26% vs 11%; *P* < 0.001), and intrauterine growth restriction (7% vs 4%; *P* = 0.040), rates of fetal and neonatal mortality were similar. Overall, patients with impaired LVF had a shorter median pregnancy duration (38 [36-39] vs 38.7 [37.4-39.7] weeks; *P* < 0.001) and babies with a lower median birth weight (2,792 [2,355-3,192.5] vs 3,020 [2,700-3,400] g; *P* < 0.001) in the presence (2,639.5 [2,357.5-3,065] vs 2,835 [2,500-3,155] g; *P* = 0.020) or absence (2,895 [2,310-3,270] vs 3,040 [2,700-3,400] g; *P* = 0.048) of beta-blocker use. Three live-born neonates died within the first 6 months of life (1.2%), all had been born prematurely. Of the 65 babies with low birth weight (26%), 52 (80%; 95% CI: 69%-88%) were born prematurely.

**Table 5:** Obstetric, Fetal, and Neonatal Outcome

	Impaired LVF (n = 251)	ROPAC Cohort (Normal LVF) (n = 5,369)	*P* Value
Obstetric outcome			
Pregnancy-induced hypertension	8 (3%, 2%-6%)	139 (3%, 2%-3%)	0.180
Pre-eclampsia, eclampsia, and HELLP	4 (2%, 1%-4%)	152 (3%, 2%-3%)	0.270
Caesarean section	141 (56%, 50%-63%)	2,290 (43%, 41%-44%)	<0.001
Emergency caesarean section	44 (18%, 14%-23%)	471 (9%, 8%-10%)	<0.001
For cardiac reasons	15 (6%, 4%-8%)	65 (1%, 1%-2%)	<0.001
Major postpartum hemorrhage	9 (4%, 2%-6%)	151 (3%, 2%-3%)	0.470
Fetal and neonatal outcome			
Fetal mortality	5 (2%, 1%-5%)	65 (1.2%, 1%-2%)	0.490
Neonatal mortality	3 (1.2%, 1%-4%)	29 (0.5%, 0.4%-0.8%)	0.180
Premature birth	67 (27%, 22%-33%)	786 (15%, 14%-16%)	<0.001
Low Apgar score	22 (9%, 6%-13%)	344 (6%, 6%-7%)	0.140
Low birth weight	65 (26%, 21%-32%)	568 (11%, 10%-11%)	<0.001
Intrauterine growth retardation	17 (7%, 5%-11%)	218 (4%, 4%-5%)	0.040
Pregnancy duration in weeks (median, IQR)	38 (36-39)	38.7 (37.4-39.7)	<0.001
Median birth weight in grams (IQR)	2,792 (2,355-3,192.5)	3,020 (2,700-3,400)	<0.001
Median birth weight (IQR) with beta-blocker use	2,639.5 (2,357.5-3,065)	2,835 (2,500-3,155)	0.020
Median birth weight (IQR) without beta-blocker use	2,895 (2,310-3,270)	3,040 (2,700-3,400)	0.048

HELLP = hemolysis, elevated liver enzymes, and low platelets; other abbreviation as in Table 1.

Values are n (%, 95% CI) unless otherwise indicated.

## Discussion

This is one of the largest studies to examine the pregnancy outcome of patients with impaired LVF in detail. In this prospective world-wide registry of 5,739 pregnancies in patients with structural and ischemic heart disease, 251 patients were identified with impaired LVF (defined as a LVEF <40%), excluding patients with a systemic right ventricle or single-ventricle morphology. Overall, at least one MACE occurred in 32% of pregnancies, which is significantly higher than in other groups of cardiac patients.^10^ NYHA functional class >2, prior atrial fibrillation, and signs of heart failure prior to pregnancy were associated with MACE in women with impaired LVF.

### Mortality

The maternal mortality rate for women with impaired LVF in our registry is 2.4%, which is significantly higher than the rates in the normal pregnant population and also than the rates in our total registry (0.6%).^10^ The 6 women who died during pregnancy suffered from acute exacerbation of heart failure or died suddenly and were diagnosed with CMP or VHD. In our study, especially patients with CMP were at high mortality risk (4.3%), which makes them more than 600 times more likely to die during pregnancy than healthy pregnant women. According to the mWHO classification, patients with an LVEF <30% should be advised against pregnancy.^6^^,^^11^ In our study, the women who died and had a known LVEF, these were all 30% or below. The high mortality rate among these patients underlines the importance of this guideline recommendation. Women with an LVEF of more than 30% but below 40%, who are included in our study, are considered to be in mWHO class III according to the guidelines. In the 2018 ESC guidelines, mWHO IV is defined as having a maternal cardiac event rate of 40% to 100%, whereas mWHO III is defined as having an event rate of 19% to 27%.^11^ With the high mortality rate and rate of MACE (almost 40%) in our study, it seems defendable to consider women with an LVEF <40% as mWHO IV. However, as we do unfortunately not know the precise LVEF of the vast majority of women, the high mortality and morbidity rates in our study could be explained by those who are currently in mWHO IV due to an LVEF of below 30%.

### Major adverse cardiac events, associations, and subgroups

Thirty-two percent of pregnancies were complicated by the occurrence of at least one MACE, of which nearly 80% required hospital admission. Heart failure is the most common complication during pregnancy and can have devastating consequences.^10^^,^^12^ Again, both women with VHD and CMP were at high risk, consistent with the current literature and risk prediction models.^7^^,^^8^^,^^10^^,^^12^ In our univariable analysis, several factors were associated with an increased risk of MACE. The prepregnancy signs of heart failure, atrial fibrillation, and NYHA functional class >II remained significantly associated in the multivariable analysis.

The patients who were in NYHA functional class III and IV prior to pregnancy the greater majority developed a MACE (79% for NYHA functional class III and 83% for NYHA functional class IV). Still, 15 percent of patients who were in NYHA functional class I prior to pregnancy developed one or more MACE during pregnancy. Clinical signs of heart failure prior to pregnancy and NYHA functional class of above II are of course somewhat inherent to impaired LVF in general. However, focusing only on the group with impaired LVF illustrates that even within this group, these parameters are still important and clinically relevant with regard to pregnancy outcome. This is important for assessing the pregnancy risk during preconception counseling of the individual patient with impaired LVF and further supports the premise that patients presenting for preconception counseling with an NYHA functional class of II or IV and clinical signs of heart failure and/or atrial fibrillation should be discouraged from becoming pregnant. Alternatively, if they are to accept the increased risk, then they should be closely monitored in a specialized center.

### Medication during pregnancy

In our cohort, many patients were using cardiac medication and anticoagulation before and during pregnancy. While patients with impaired LVF compose a group in which cardiac medications are regularly prescribed, pregnancy poses pharmacotherapeutical dilemma.^13^ In our cohort, beta-blockers and antiplatelet therapy were used more frequently during than before pregnancy, possibly for prophylaxis, worsening symptoms, or the detection of signs of heart failure on physical examination. The rate of ACE inhibitor use during pregnancy (3%) is remarkable but comparable to previous studies, which state that 32% of women with heart disease use medications at some time during pregnancy and that ACE inhibitors were used in 2.8% of pregnancies.^14^ As the fetotoxic effects of ACE inhibitors are well known,^15^ the rate of ACE inhibitor use in our study could reflect the severity of disease in these women and the difficult balance between maternal health and fetal risks. Ideally, optimization with medication, (surgical) interventions, or cardiac rehabilitation should be undertaken prior to conception to optimize maternal health and minimize the need for potentially teratogenetic drugs.

### Fetal and obstetric outcomes

Interestingly, we found no evidence of an increased risk of (pre-)eclampsia or pregnancy-induced hypertension when compared to the normal population. At first sight, this seems to contradict previous data, where pre-eclampsia was associated with heart failure during pregnancy.^12^ However, pre-eclampsia increases the risk of pulmonary edema and is associated with peripartum CMP in its own right, suggesting that the link with heart failure may be secondary to the occurrence of pre-eclampsia, rather than women with heart failure being at greater risk of pre-eclampsia. Consistent with this possibility, pregnancy-associated hypertensive disorders are more often related to heart failure with preserved LVEF.^16^

CS rates were high in our cohort, perhaps related to the pregnancy guidelines which state that the presence of heart failure is an indication for CS. Equally, this situation would usually be classified as an emergency, that is unplanned, CS and only 6% of patients required an emergency CS for cardiac reasons. Similarly, the high rates of preterm delivery (27%) could be explained, at least in part, by planned preterm delivery in women with significant exacerbation of heart failure, but again this should have been classified as an emergency CS or induction for cardiac indications. Alternatively, the decision to delivery early may have been made to shorten the maternal exposure to the impact of pregnancy. The increased rates of low birth weight (26%) could be related to the high rates of preterm delivery, but the frequent use of beta-blockers during pregnancy may also have played a role.^14^ Interestingly, no associations with adverse obstetric and fetal outcome were identified in our study.

### Study limitations

This study, as are other registries, has limitations due to the availability of information on the past history. While our cohort is the largest to date, data on systemic ventricular function were missing in 27% of all 5,739 included pregnancies. Data on 6 months follow-up were not available in 27% of the impaired LVF patients.

The prospective character of this study should in theory prevent selection bias. However, the centers participated on a voluntary basis and they selected which patients to include and therefore selection bias should always be kept in mind when interpreting the results. Another limitation is that we did not correct for between-center heterogeneity caused by the large number of included centers. Furthermore, we did not account for potential center-level random effects, which could impact the generalizability of the findings. This study includes global data and, therefore, reflects global patient characteristics, enhancing the generalizability of the findings. Moreover, ROPAC includes pregnancies and not patients. Multiple pregnancies from one woman could have been included. This could induce bias as a woman who becomes pregnant more than once will usually have less severe disease.

It is a clear limitation that the definition of LVEF on echo was not predefined but left to the clinical practice of the treating physician. Also, by selecting patients with LVEF <40%, we sought to identify the group of patients with impaired LVF. Specific, detailed echocardiographic (or advanced imaging) data on LVEF and LVF, however, were not available in many patients (79%). Besides, the definition of heart failure had changed during the extend of this registry. Therefore, although this creates heterogeneity, we decided the diagnosis should be determined by the treating physician.

Furthermore, it is important to note that the multivariable analyses in this study were exploratory in nature. Therefore, the results should be interpreted as associations rather than causal relationships. Despite these limitations, this registry included a large number of patients with impaired LVF, providing clinically important information on the pregnancy outcomes in this group.

## Conclusions

Women with impaired LVF are at increased risk of maternal mortality and maternal and fetal morbidity, particularly heart failure, tachyarrhythmias, and premature delivery with low birth weight. Thirty-two percent of patients suffered from one or more cardiac events during pregnancy or in the first 6 months postpartum. Prepregnancy signs of heart failure, atrial fibrillation, and NYHA functional class > II were associated with a poor cardiac outcome. Women with impaired LVF should undergo preconception counseling, and the potential consequences of pregnancy should be discussed with them. Our results emphasize the need for prepregnancy treatment optimization and intensive monitoring not only during pregnancy but also after delivery.

Perspectives **COMPETENCY IN MEDICAL KNOWLEDGE:** This prospective registry-based study highlights the high maternal and fetal risks associated with pregnancy in women with impaired LVF (LVEF <40%), including a 32% cardiac event rate and 2.4% mortality. Women with prepregnancy heart failure symptoms, atrial fibrillation, or NYHA functional class > II are at especially high risk and should receive thorough preconception counseling and close multidisciplinary monitoring. Findings support considering women with LVEF <40% as mWHO class IV.

**TRANSLATIONAL OUTLOOK:** Translational challenges include incomplete echocardiographic data, lack of fetal risk associations, and variable medication use. Further research should focus on refining risk stratification, optimizing care strategies, and improving outcomes in resource-limited settings.

## Funding support and author disclosures

Funding from “Zabawas Foundation” and “De Hoop Foundation” in addition to the support from EORP is greatly acknowledged. Since the start of EORP, the following companies have supported the program: Abbott Vascular Int (2011-2021), Amgen Cardiovascular (2009-2018), AstraZeneca (2014-2021), Bayer AG (2009-2018), Boehringer Ingelheim (2009-2019), Boston Scientific (2009-2012), The Bristol Myers Squibb and Pfizer Alliance (2011-2019), Daiichi Sankyo Europe GmbH (2011-2020), The Alliance Daiichi Sankyo Europe GmbH and Eli Lilly and Company (2014-2017), Edwards (2016-2019), Gedeon Richter Plc. (2014-2016), Menarini Int. Op. (2009-2012), MSD-Merck & Co (2011-2014), Novartis Pharma AG (2014-2020), ResMed (2014-2016), Sanofi (2009-2011), and SERVIER (2009-2021). The authors have reported that they have no relationships relevant to the contents of this paper to disclose.

## References

[1] Lloyd-Jones D.M., Larson M.G., Leip E.P. (2002). Lifetime risk for developing congestive heart failure: the Framingham Heart Study. Circulation.

[2] Yusuf S.W., Ilias-Khan N.A., Durand J.B. (2011). Chemotherapy-induced cardiomyopathy. Expert Rev Cardiovasc Ther.

[3] Capeless E.L., Clapp J.F. (1989). Cardiovascular changes in early phase of pregnancy. Am J Obstet Gynecol.

[4] Katz R., Karliner J.S., Resnik R. (1978). Effects of a natural volume overload state (pregnancy) on left ventricular performance in normal human subjects. Circulation.

[5] Lund C.J., Donovan J.C. (1967). Blood volume during pregnancy. Significance of plasma and red cell volumes. Am J Obstet Gynecol.

[6] Regitz-Zagrosek V., Blomstrom Lundqvist C., Borghi C. (2011). ESC guidelines on the management of cardiovascular diseases during pregnancy: the Task Force on the Management of Cardiovascular Diseases during Pregnancy of the European Society of Cardiology (ESC). Eur Heart J.

[7] Siu S.C., Sermer M., Colman J.M. (2001). Prospective multicenter study of pregnancy outcomes in women with heart disease. Circulation.

[8] Roos-Hesselink J.W., Ruys T.P., Stein J.I. (2013). Outcome of pregnancy in patients with structural or ischaemic heart disease: results of a registry of the European Society of Cardiology. Eur Heart J.

[9] Tranquilli A.L., Brown M.A., Zeeman G.G., Dekker G., Sibai B.M. (2013). The definition of severe and early-onset preeclampsia. Statements from the International Society for the Study of Hypertension in Pregnancy (ISSHP). Pregnancy Hypertens.

[10] van Hagen I.M., Boersma E., Johnson M.R. (2016). Global cardiac risk assessment in the registry of pregnancy and cardiac disease: results of a registry from the European Society of Cardiology. Eur J Heart Fail.

[11] Regitz-Zagrosek V., Roos-Hesselink J.W., Bauersachs J. (2018). 2018 ESC guidelines for the management of cardiovascular diseases during pregnancy. Eur Heart J.

[12] Ruys T.P., Roos-Hesselink J.W., Hall R. (2014). Heart failure in pregnant women with cardiac disease: data from the ROPAC. Heart.

[13] Pieper P.G. (2015). Use of medication for cardiovascular disease during pregnancy. Nat Rev Cardiol.

[14] Ruys T.P., Maggioni A., Johnson M.R. (2014). Cardiac medication during pregnancy, data from the ROPAC. Int J Cardiol.

[15] Barr M. (1994). Teratogen update: angiotensin-converting enzyme inhibitors. Teratology.

[16] Ntusi N.B., Badri M., Gumedze F., Sliwa K., Mayosi B.M. (2015). Pregnancy-associated heart failure: a comparison of clinical presentation and outcome between hypertensive heart failure of pregnancy and idiopathic peripartum cardiomyopathy. PLoS One.

