# Supplementary material for: Pregnancy in Women With Impaired Left Ventricular Function: Data From ROPAC
Source: JACC Adv. 2026 Feb 25;5(3):102605. doi: 10.1016/j.jacadv.2026.102605 (PMC12955112; doi:10.1016/j.jacadv.2026.102605)
Supplement: Supplementary data 1 [file mmc1.docx]

**Supplementary Appendix**

1. EORP Oversight Committee, ROPAC Executive Committee en List of Centers and ROPAC Investigators
2. Supplementary Tables
3. Supplementary Figures
4. **EORP Oversight Committee, ROPAC Executive Committee en List of Centers and ROPAC Investigators**

**EORP Oversight Committee**

Christopher Peter Gale, Chair, GB, Branko Beleslin, RS, Andrzej Budaj, PL, Ovidiu Chioncel, RO, Nikolaos Dagres, DE, Nicolas Danchin, FR, David Erlinge, SE, Jonathan Emberson, GB, Michael Glikson, IL, Alastair Gray, GB, Meral Kayikcioglu, TR, Aldo Maggioni, IT, Klaudia Vivien Nagy, HU, Aleksandr Nedoshivin, RU, Anna-Sonia Petronio, IT, Jolien Roos-Hesselink, NL, Lars Wallentin, SE, Uwe Zeymer, DE.

**Executive Committee:**

Roger Hall GB (Co-Chair), Jolien Roos-Hesselink NL (Co-Chair), Joerg Stein, AT, William Anthony Parsonage, AU, Werner Budts, BE, Julie De Backer, BE, Jasmin Grewal, CA, Ariane Marelli, CA, Harald Kaemmerer, DE, Guillaume Jondeau, FR, Mark Johnson, GB, Aldo P. Maggioni, IT, Luigi Tavazzi, IT, Ulf Thilen, SE, Uri Elkayam, US, Catherine Otto, US, Karen Sliwa, ZA.

**CENTRES**

**ARGENTINA** -   Buenos Aires: A. Aquieri, A. Saad, H. Ruda Vega, J. Hojman, J.M. Caparros, M. Vazquez Blanco,    **AUSTRALIA** -   Elizabeth Vale: M. Arstall, C.M. Chung, G. Mahadavan, E. Aldridge, M. Wittwer, Y.Y. Chow,   Herston: W.A. Parsonage, K. Lust,   New Lambton Heights: N. Collins, G. Warner, R. Hatton, A. Gordon, E. Nyman,    **AUSTRIA** -   Innsbruck: J. Stein , E. Donhauser ,   Vienna: H. Gabriel,    **AZERBAIJAN** -   Baku: A. Bahshaliyev, F. Guliyev, I. Hasanova, T. Jahangirov, Z. Gasimov,    **BANGLADESH** -   Dhaka: A. Salim, C.M. Ahmed, F. Begum, M.H. Hoque, M. Mahmood, M.N. Islam, P.P. Haque, S.K. Banerjee, T. Parveen,    **BELGIUM** -   Brussels: M. Morissens,   Gent: J. De Backer, L. Demulier, M. de Hosson,   Leuven: W. Budts, M. Beckx,    **BOSNIA AND HERZEGOVINA** -   Banja Luka: M. Kozic, M. Lovric, T. Kovacevic-Preradovic,    **BULGARIA** -   Sofia: N. Chilingirova, P. Kratunkov,    **CANADA** -   Edmonton: N. Wahab, S. McLean,   Hamilton, Ontario: E. Gordon , L. Walter ,   Montreal: A. Marelli , A. R. Montesclaros,    **COLOMBIA**  -   Medellin: G. Monsalve, C. Rodriguez, F. Balthazar, V. Quintero, W. Palacio, L.A. Mejía Cadavid,   Medellin: E. Munoz Ortiz, F. Fortich Hoyos, E. Arevalo Guerrero, J. Gandara Ricardo, J. Velasquez Penagos,    **CZECH REPUBLIC** -   Hradec Kralove: Z. Vavera,   Prague: J. Popelova ,    **DENMARK** -   Copenhagen: N. Vejlstrup, L. Grønbeck, M. Johansen, A. Ersboll,    **EGYPT** -   Alexandria: Y. Elrakshy,   Assiut: K. Eltamawy , M. Gamal Abd-El Aziz,   Benha : A. El Nagar, H. Ebaid, H. Abo Elenin, M. Saed, S. Farag, W. Makled,   Cairo: K. Sorour, Z. Ashour, G. El-Sayed, M. Abdel Meguid Mahdy,   Minia: N. Taha, A. Dardeer, M. Shabaan,   Zagazig: A. Saad, M. Ali,    **FRANCE** -   Nice: P. Moceri,   Paris: G. Duthoit, M. Gouton, J. Nizard, L. Baris,   Paris: S. Cohen, M. Ladouceur, L. Baris, D. Khimoud,   Paris: B. Iung ,    **GERMANY** -   Berlin: F. Berger, A. Olsson,   Bonn: U. Gembruch, W.M. Merz, E. Reinert, S. Clade, Y. Kliesch,   Essen: C. Wald ,   Hamburg: C. Sinning, R. Kozlik-Feldmann, S. Blankenberg, E. Zengin-Sahm, G. Mueller, M. Hillebrand, P. Hauck, Y. von Kodolitsch, N. Zarniko,   Muenster: H. Baumgartner, R. Schmidt, A. Hellige,   Munich: O. Tutarel, H. Kaemmerer, B. Kuschel, N. Nagdyman,   Oldenburg: R. Motz ,    **GEORGIA** -   Tbilisi: D. Maisuradze, **GREECE** -   Athens: A. Frogoudaki, E. Iliodromitis, M. Anastasiou-Nana,   Marousi, Athens: D. Triantafyllis, G. Bekiaris,   Thessaloniki: H. Karvounis, G. Giannakoulas, D. Ntiloudi, S.A. Mouratoglou,    **HUNGARY** -   Budapest: A. Temesvari, H. Balint, D. Kohalmi,   Budapest: B. Merkely, C. Liptai,   Szeged: A. Nemes, T. Forster, A. Kalapos, K. Berek, K. Havasi, N. Ambrus,    **INDIA** -   Karad: A. Shelke, R. Kawade, S. Patil,    **INDONESIA** -   Bandung: E. Martanto, T.M. Aprami, A. Purnomowati, C.J. Cool, M. Hasan, R. Akbar, S. Hidayat, T.I. Dewi, W. Permadi, D.A. Soedarsono,    **IRAN**  -   Tehran: M.M. Ansari-Ramandi, N. Samiei, A. Tabib, F. Kashfi, S. Ansari-Ramandi, S. Rezaei,    **IRAQ** -   Baghdad: H. Ali Farhan, A. Al-Hussein, G. Al-Saedi, G. Mahmood, I.F. Yaseen, L. Al-Yousuf, M. AlBayati, S. Mahmood, S. Raheem, T. AlHaidari, Z. Dakhil,    **IRELAND** -   Dublin: P. Thornton, J. Donnelly, M. Bowen,    **ISRAEL** -   Beer Yakov: A. Blatt ,   Beer Yakov: A. Blatt, G. Elbaz-Greener,   Hadera: A. Shotan,   Haifa: S. Yalonetsky,   Rehovot: S. Goland, M. Biener,    **ITALY** -   Bologna: G. Egidy Assenza, M. Bonvicini, A. Donti, A. Bulgarelli, D. Prandstraller,   Bolzano: C. Romeo, R. Crepaz,   Brescia: E. Sciatti, M. Metra, R. Orabona,   Massa: L. Ait Ali, P. Festa,   Milano: V. Fesslova ,   Milano: C. Bonanomi, M. Calcagnino, F. Lombardi, A.M. Colli, M.W. Ossola, C. Gobbi, E. Gherbesi, L. Tondi, M. Schiavone, M. Squillace,   Palermo: M.G. Carmina,   Torino: A. Maina, C. Macchi, E. Gollo, F.M. Comoglio, N. Montali, P. Re, R. Bordese, T. Todros, V. Donvito, W. Grosso Marra,   Trieste: G. Sinagra, B. D'Agata Mottolese, M. Bobbo, V. Gesuete, S. Rakar, F. Ramani,    **JAPAN** -   Chiba: K. Niwa ,    **KAZAKHSTAN** -   Almaty: D. Mekebekova, A. Mussagaliyeva, T. Lee,    **KYRGYZSTAN**  -   Bishkek: E. Mirrakhimov, S. Abilova, E. Bektasheva, K. Neronova, O. Lunegova,    **LITHUANIA** -   Kaunas: R. Žaliūnas, R. Jonkaitienė, J. Petrauskaitė,   Vilnius: A. Laucevicius, D. Jancauskaite, L. Lauciuviene, L. Gumbiene, L. Lankutiene, S. Glaveckaite, M. Laukyte, S. Solovjova, V. Rudiene,    **MALAYSIA**  -   Kuala Lumpur: K.H. Chee, C.C-W. Yim, H.L. Ang, R. Kuppusamy, T. Watson,    **MALTA** -   Birkirkara: M. Caruana,    **NORWAY** -   Oslo: M-E. Estensen,    **PAKISTAN** -   Rawalpindi: M.G.A. Mahmood Kayani, R. Munir,    **POLAND**  -   Bialystok: A. Tomaszuk-Kazberuk, B. Sobkowicz, J. Przepiesc,   Krakow: A. Lesniak-Sobelga, L. Tomkiewicz-Pajak, M. Komar, M. Olszowska, P. Podolec, S. Wisniowska-Smialek,   Lodz: M. Lelonek, U. Faflik, A. Cichocka-Radwan,  Poznan: K. Plaskota, O. Trojnarska,    **PORTUGAL** -Coimbra: N. Guerra ,   Lisboa: L. de Sousa,   Porto: C. Cruz, V. Ribeiro, **REPUBLIC OF MACEDONIA** -   Skopje: S. Jovanova,    **ROMANIA** -   Bucharest: V. Petrescu ,   Bucharest: R. Jurcut, C. Ginghina, I. Mircea Coman, M. Musteata,    **RUSSIA** -   Belgorod: O. Osipova, T. Golivets, I. Khamnagadaev, O. Golovchenko, A. Nagibina, I. Ropatko,   Izhevsk: I.R. Gaisin, L. Valeryevna Shilina,   Moscow: N. Sharashkina,   Saint- Petersburg: E. Shlyakhto, O. Irtyuga, O. Moiseeva, E. Karelkina, I. Zazerskaya, A. Kozlenok, I. Sukhova,    **SERBIA** -   Belgrade: L. Jovovic ,    **SLOVENIA** -   Ljubljana: K. Prokšelj, M. Koželj,    **SOMALILAND** -   Hargeisa: A.O. Askar, A.A. Abdilaahi, M.H. Mohamed, A.M. Dirir,    **SOUTH** **AFRICA** -   Cape Town: K. Sliwa,   Houghton: P. Manga,    **SPAIN** -   Barcelona: A. Pijuan-Domenech, L. Galian-Gay, P. Tornos, M.T. Subirana,   Barcelona: M. T.  Subirana ,   Bilbao: N. Murga,   Madrid: J. M. Oliver ,   Madrid: B. Garcia-Aranda Dominguez, I. Hernandez Gonzalez, J.F. Delgado Jimenez, P. Escribano Subias,    **SUDAN** -   Khartoum: A. Elbushi, A. Suliman, K. Jazzar, M. Murtada, N. Ahamed,    **SWEDEN**  -   Göteborg: M. Dellborg, E. Furenas, M. Jinesjo, K. Skoglund, P. Eriksson, T. Gilljam,   Lund: U. Thilen,    **SWITZERLAND** -   Basel: D. Tobler,   Bern: K. Wustmann, F. Schwitz, M. Schwerzmann,   Lausanne: T. Rutz, J. Bouchardy,   Zurich: M. Greutmann, B.M. Santos Lopes, L. Meier, M. Arrigo,    **THE NETHERLANDS** -   Amsterdam: K. de Boer, T. Konings,   Enschede: E. Wajon, L.J. Wagenaar,   Geldrop: P. Polak ,   Groningen: E.PG. Pieper,   Rotterdam: J. Roos-Hesselink, L. Baris, I. van Hagen, H. Duvekot, J.M.J. Cornette, The Hague: C. De Groot,   Utrecht: C. van Oppen,    **TURKEY** -   Istanbul: L. Sarac, O. Batukan Esen, S. Catirli Enar,   **UGANDA** -   Kampala: C. Mondo, P. Ingabire, B. Nalwanga, T. Semu,    **UNITED ARAB EMIRATES** -   Abu Dhabi: B.T. Salih, W.A.R. Almahmeed, S. Wani, F.S. Mohamed Farook,   Al Ain, Abu Dhabi: F. Gerges, A.M. Komaranchath, F. Al bakshi,   Dubai: A. Al Mulla, A.H. Yusufali, E.I. Al Hatou, N. Bazargani, F. Hussain,    **UNITED KINGDOM** -   Birmingham: L. Hudsmith, P. Thompson, S. Thorne, S. Bowater,   Buckinghamshire: A. Money-Kyrle, P. Clifford, P. Ramrakha, S. Firoozan, J. Chaplin, N. Bowers,   Coventry: D. Adamson,   London: F. Schroeder, R. Wendler, S. Hammond, London: P. Nihoyannopoulos ,   Norwich Norfolk: R. Hall , L. Freeman ,   Southampton: G. Veldtman , J. Kerr , L. Tellett ,    **UNITED STATES** -   Boston: N. Scott, A.B. Bhatt, D. DeFaria Yeh, M.A. Youniss, M. Wood, A.A. Sarma, S. Tsiaras, A. Stefanescu, J.M. Duran, L. Stone,   Cleveland: D.S. Majdalany, J. Chapa,   Detroit: K. Chintala , P. Gupta ,   Hershey, PA: J. Botti , J. Ting, W. R. Davidson,   Lexington, Kentucky: G. Wells, D. Sparks,   Mineola, NY: V. Paruchuri, K. Marzo, D. Patel,   Minneapolis: W. Wagner, S.N. Ahanya, L. Colicchia, T. Jentink, K. Han, M. Loichinger, M. Parker, W. Wagner, C. Longtin,   Omaha: A. Yetman, K. Erickson, J. Cramer, S. Tsai, B. Fletcher, S. Warta,   Phoenix: C. Cohen, C. Lindblade, R. Puntel, K. Nagaran, N. Croft,   Seattle: M. Gurvitz , C. Otto,   Stanford, CA: C. Talluto , D. Murphy , M. G. Perlroth

1. **Supplementary tables**

**Table S1** Effect estimates after multiple imputation for pre-pregnancy cardiac history variables in women with reduced left ventricular function

| **Pre-pregnancy history** | **Missing, n (%)** | **OR** | **95% CI** | **P-value** |
| --- | --- | --- | --- | --- |
| Age | 20 (17.4%) | 0.827 | 0.641-1.067 | 0.143 |
| Nulliparity | 0 (0%) | 8.222 | 0.887-76.252 | 0.064 |
| NYHA > 2 | 0 (0%) | 11.192 | 1.707-7.386 | 0.012 |
| Prior medication | 0 (0%) | 1.057 | 0.113-9.923 | 0.961 |
| Anticoagulation | 0 (0%) | 1.214 | 0.194-7.576 | 0.836 |
| Hypertension | 0 (0%) | 1.197 | 0.127-11.319 | 0.875 |
| Heart failure | 1 (0.9%) | 1.762 | 0.280-11.071 | 0.546 |

CI, confidence interval; MICE, multivariate imputation by chained equations*;* NYHA*,* New York Heart Association; OR, odds ratio

**Table S2** Effect estimates from complete-case sensitivity analysis for pre-pregnancy cardiac history variables in women with reduced left ventricular function

| **Pre-pregnancy history** | **OR** | **95% CI** | **P-value** |
| --- | --- | --- | --- |
| Age | 0.827 | 0.641-1.067 | 0.143 |
| Nulliparity | 8.222 | 0.887-76.252 | 0.064 |
| NYHA > 2 | 11.192 | 1.707-7.386 | 0.012 |
| Prior medication | 1.057 | 0.113-9.923 | 0.961 |
| Anticoagulation | 1.214 | 0.194-7.576 | 0.836 |
| Hypertension | 1.197 | 0.127-11.319 | 0.875 |
| Heart failure | 1.756 | 0.279-11.031 | 0.548 |

CI, confidence interval; MICE, multivariate imputation by chained equations; NYHA, New York Heart Association; OR, odds ratio

| **Table S3** Cardiac medication before and during pregnancy in 251 women with reduced and normal LVF | | | | |
| --- | --- | --- | --- | --- |
| **Reduced LVF** | | | **Normal LVF** | |
|  | **Before pregnancy (%)** | **During pregnancy (%)** | **Before pregnancy (%)** | **During pregnancy (%)** |
| Beta blocker | 87 (35%) | 118 (53%) | 358 (7%) | 629 (12%) |
| Diuretics | 9 (4%) | 14 (6%) | 39 (1%) | 46 (1%) |
| ACE-inhibitor | 21 (8%) | 8 (3%) | 56 (1%) | 15 (0.3%) |
| Vitamin-K-antagonists | 27 (11%) | 25 (10%) | 367 (7%) | 334 (6%) |
| Antiplatelet therapy | 20 (8%) | 28 (11%) | 214 (4%) | 324 (6%) |

ACE, angiotensin converting enzyme; LVF, left ventricular function

| **Table S4** Pre-pregnancy predictors of MACE | | | |
| --- | --- | --- | --- |
| **Univariable** | **Odds ratio** | **P-value** | **95% CI** |
| Age | 0.98 | 0.470 | 0.936-1.031 |
| Signs of heart failure | 5.46 | **<0.001** | 3.000-9.936 |
| Hypertension | 1.39 | 0.390 | 0.653-2.937 |
| Prior atrial fibrillation | 6.02 | **0.009** | 1.555-23.290 |
| Emerging country | 1.78 | **0.030** | 1.051-3.001 |
| Nulliparity | 0.82 | 0.460 | 0.481-1.393 |
| NYHA class < 2 | 11.25 | **<0.001** | 4.653-27.173 |
| Prior cardiac medication | 0.94 | 0.820 | 0.549-1.609 |
| Multiple gestation | 1.33 | 0.550 | 0.526-3.381 |
| **Multivariable** |  |  |  |
| Signs of heart failure | 2.67 | **0.009** | 1.284-5.571 |
| NYHA class > 2 | 6.06 | **<0.001** | 2.210-16.632 |
| Prior atrial fibrillation | 6.32 | **0.013** | 3.015-13.260 |

MACE, major adverse cardiac events; NYHA, New York Health Association

| **Table S5** Baseline characteristics of pregnant cardiomyopathy patients with and without impaired LVF | | | | | | |
| --- | --- | --- | --- | --- | --- | --- |
|  | **CMP with impaired LVF**  N = 115 | | **CMP with normal LVF**  N = 323 | **P-value** | |  |
| **Demographics** |  |  | | |  | |
| Mean age in years (SD) | 31.4 (5.6) | 30.9 (5.9) | | |  | |
| Nulliparity | 40 (35%) | 128 (40%) | | | 0.360 | |
| Emerging country | 66 (57%) | 120 (37%) | | | **<0.001** | |
| **Diagnosis details** |  |  | | |  | |
| Dilated cardiomyopathy | 55 (48%) | 31 (10%) | | | **<0.001** | |
| Hypertrophic cardiomyopathy | 5 (4%) | 88 (27%) | | | **<0.001** | |
| Peripartum cardiomyopathy in history | 25 (22%) | 34 (11%) | | | **0.002** | |
| Chemotherapy-induced cardiomyopathy | 7 (6%) | 3 (1%) | | | 0.080 | |
| Left ventricular non-compaction | 11 (10%) | 9 (3%) | | | **0.046** | |
| Arrhythmogenic right ventricular cardiomyopathy | 3 (3%) | 10 (3%) | | | 0.380 | |
| Myocarditis | 6 (5%) | 15 (5%) | | | 0.590 | |
| **Pre-pregnancy history** |  |  | | |  | |
| Current smoking | 9 (8%) | 20 (6%) | | | 0.310 | |
| Hypertension | 20 (17%) | 35 (11%) | | | **0.030** | |
| Heart failure | 32 (28%) | 48 (15%) | | | **0.003** | |
| Atrial fibrillation | 0 (0%) | 3 (1%) | | | 0.300 | |
| Angina pectoris | 6 (5%) | 15 (5%) | | | 0.940 | |
| Diabetes mellitus | 1 (1%) | 5 (1.5%) | | | 0.220 | |
| NYHA class I | 42 (37%) | 203 (63%) | | | **<0.001** | |
| NYHA class II | 51(44%) | 85 (26%) | | | **<0.001** | |
| NYHA class III | 10 (9%) | 17 (5%) | | | 0.190 | |
| NYHA class IV | 6 (5%) | 3 (1%) | | | **0.005** | |
| Prior intervention | 11 (10%) | 48 (15%) | | | 0.240 | |
| Prior medication | 91 (79%) | 146 (45%) | | | **<0.001** | |
| Beta blocker | 56 (49%) | 53 (16%) | | | **<0.001** | |
| Diuretics | 6 (5%) | 4 (1%) | | | **0.010** | |
| ACE-inhibitor | 16 (14%) | 6 (2%) | | | **<0.001** | |
| Anticoagulation | 41 (36%) | 51 (16%) | | | **<0.001** | |

ACE, angiotensin converting enzyme; CMP, cardiomyopathy; LVF, left ventricular dysfunction; NYHA, New York Heart Association

| **Table S6** Cardiovascular outcome in pregnant cardiomyopathy patients | | | | | |
| --- | --- | --- | --- | --- | --- |
|  | **Total CMP cohort**  N = 438 | **Impaired LVF**  N = 115 | **Normal**  **LVF**  N = 323 | **P-value** | |
| MACE | 163 (38%) | 45 (39%) | 118 (37%) | 0.620 |  |
| Maternal mortality | 8 (1.8%) | 5 (4.3%) | 3 (0.9%) | **0.020** |  |
| Hospital admission for cardiac reasons | 107 (24%) | 34 (30%) | 27 (23%) | 0.140 |  |
| Heart failure episode during pregnancy | 122 (28%) | 36 (31%) | 86 (27%) | 0.340 |  |
| Heart failure episode post-partum | 29 (7%) | 9 (8%) | 20 (6%) | 0.550 |  |
| Ventricular tachyarrhythmia | 32 (7%) | 9 (8%) | 23 (7%) | 0.800 |  |
| Atrial fibrillation or flutter | 9 (2%) | 2 (2%) | 7 (2%) | 0.780 |  |
| Endocarditis | 1 (0.2%) | 1 (1%) | 0 (0%) | 0.090 |  |
| Aortic dissection | 0 (0%) | 0 (0%) | 0 (0%) | - |  |
| Ischemic coronary event | 1 (0.2%) | 1 (1%) | 0 (0%) | 0.090 |  |
| Other thrombo-embolic events | 4 (1%) | 4 (3.5%) | 0 (0%) | **0.001** |  |

P-values were calculated between the group with impaired LV function and normal LV function

CMP, cardiomyopathy; LVF, left ventricular function; MACE, adverse cardiac events

| **Table S7** Baseline characteristics of pregnant congenital heart disease patients with and without impaired LVF | | | | |
| --- | --- | --- | --- | --- |
|  | **CHD with impaired LVF**  N = 62 |  | **CHD with normal LVF**  N = 3114 | **P-value** |
| **Demographics** |  | |  |  |
| Mean age in years (SD) | 28.8 (5.4) | | 29.0 (5.4) | 0.690 |
| Nulliparity | 36 (58%) | | 1598 (51%) | 0.540 |
| Emerging country | 20 (32%) | | 900 (29%) | 0.560 |
| **Diagnosis details** |  | |  |  |
| ASD/VSD/AVSD | 17 (27%) | | 1110 (36%) | 0.180 |
| Fallot | 11 (18%) | | 415 (13%) | 0.310 |
| TGA arterial switch | 2 (3%) | | 177 (6%) | 0.410 |
| Pulmonary atresia | 2 (3%) | | 30 (1%) | 0.080 |
| Patent ductus arteriosus | 2 (3%) | | 69 (2%) | 0.590 |
| Pulmonary vein abnormality | 3 (5%) | | 30 (1%) | **0.003** |
| Other CHD | 25 (40%) | | 1283 (41%) | 0.890 |
| **Pre-pregnancy history** |  | |  |  |
| Current smoking | 2 (3%) | | 126 (4%) | 0.690 |
| Hypertension | 4 (7%) | | 177 (6%) | 0.970 |
| Heart failure | 10 (16%) | | 196 (6%) | **0.001** |
| Atrial fibrillation | 1 (2%) | | 15 (0.5%) | 0.210 |
| Angina pectoris | 2 (3%) | | 49 (2%) | 0.570 |
| Diabetes mellitus | 0 (0%) | | 41 (1%) | 0.260 |
| NYHA class I | 43 (69%) | | 2474 (79%) | **0.050** |
| NYHA class II | 12 (19%) | | 522 (17%) | 0.590 |
| NYHA class III | 5 (8%) | | 48 (2%) | **<0.001** |
| NYHA class IV | 0 (0%) | | 4 (0.1%) | 0.780 |
| Prior intervention | 47 (76%) | | 2124 (68%) | 0.420 |
| Prior medication | 22 (36%) | | 651 (21%) | **0.005** |
| Beta blocker | 10 (16%) | | 139 (5%) | **<0.001** |
| Diuretics | 1 (2%) | | 14 (0.4%) | 0.260 |
| ACE-inhibitor | 1 (2%) | | 19 (1%) | 0.320 |
| Anticoagulation | 12 (21%) | | 402 (13%) | 0.060 |

ACE, angiotensin converting enzyme; ASD, atrial septal defect; AVSD, atrioventricular septal defect; CHD, congenital heart disease; LVF, left ventricular dysfunction; NYHA, New York Heart Association; VSD, ventricular septal defect

| **Table S8** Cardiovascular outcome in pregnant congenital heart disease patients | | | | | |
| --- | --- | --- | --- | --- | --- |
|  | **Total CHD cohort**  N = 3176 | **Impaired LVF**  N = 115 | **Normal LVF**  N = 323 | **P-value** | |
| MACE | 256 (8%) | 13 (21%) | 2438 (8%) | **<0.001** |  |
| Maternal mortality | 9 (0.3%) | 0 (0%) | 9 (0.3%) | 0.670 |  |
| Hospital admission for cardiac reasons | 267 (8%) | 9 (15%) | 258 (8%) | 0.080 |  |
| Heart failure episode during pregnancy | 192 (6%) | 12 (19%) | 180 (6%) | **<0.001** |  |
| Heart failure episode post-partum | 21 (1%) | 1 (2%) | 20 (1%) | 0.350 |  |
| Ventricular tachyarrhythmia | 35 (1%) | 2 (3%) | 33 (1%) | 0.110 |  |
| Atrial fibrillation or flutter | 31 (1%) | 2 (3%) | 29 (1%) | 0.070 |  |
| Endocarditis | 19 (0.6%) | 0 (%) | 19 (0.6%) | 0.540 |  |
| Aortic dissection | 0 (0%) | 0 (0%) | 0 (0%) | - |  |
| Ischemic coronary event | 0 (0%) | 0 (0%) | 0 (0%) | - |  |
| Other thrombo-embolic events | 34 (1%) | 2 (3%) | 32 (1%) | 0.890 |  |

P-values were calculated between the group with impaired LV function and normal LV function

CHD, congenital heart disease; LVF, left ventricular function; MACE, adverse cardiac events

| **Table S9** Baseline characteristics of valvular heart disease patients with and without impaired LVF | | | | |
| --- | --- | --- | --- | --- |
|  | **VHD with impaired LVF**  N = 55 |  | **VHD with normal LVF**  N = 1593 | **P-value** |
| **Demographics** |  | |  |  |
| Mean age in years (SD) | 31.0 (5.6) | | 29.7 (5.9) | 0.100 |
| Nulliparity | 16 (29%) | | 540 (34%) | 0.670 |
| Emerging country | 40 (73%) | | 1003 (63%) | 0.140 |
| **Diagnosis details** |  | |  |  |
| Mitral stenosis | 15 (28%) | | 273 (17%) | 0.050 |
| Mitral regurgitation | 18 (33%) | | 480 (30%) | 0.680 |
| Mitral stenosis and regurgitation | 15 (28%) | | 263 (17%) | 0.040 |
| Aortic stenosis | 1 (2%) | | 137 (9%) | 0.070 |
| Aortic regurgitation | 2 (4%) | | 146 (9%) | 0.160 |
| Aortic stenosis and regurgitation | 1 (2%) | | 69 (4%) | 0.360 |
| Pulmonary valve stenosis/regurgitation | 2 (4%) | | 100 (6%) | 0.420 |
| Other | 1 (2%) | | 125 (8%) | 0.270 |
| **Pre-pregnancy history** |  | |  |  |
| Current smoking | 2 (4%) | | 36 (2%) | 0.760 |
| Hypertension | 7 (13%) | | 68 (4%) | **0.003** |
| Heart failure | 19 (35%) | | 254 (16%) | **<0.001** |
| Atrial fibrillation | 10 (18%) | | 76 (5%) | **<0.001** |
| Angina pectoris | 3 (6%) | | 56 (4%) | 0.260 |
| Diabetes mellitus | 0 (0%) | | 21 (1%) | 0.560 |
| Mechanical prosthesis | 15 (27%) | | 267 (17%) | **0.040** |
| Bioprosthesis | 1 (2%) | | 61 (4%) | 0.440 |
| NYHA class I | 24 (44%) | | 1054 (66%) | **0.001** |
| NYHA class II | 17 (31%) | | 424 (27%) | 0.480 |
| NYHA class III | 12 (22%) | | 75 (5%) | **<0.001** |
| NYHA class IV | 0 (0%) | | 11 (1%) | 0.540 |
| Prior medication | 42 (76%) | | 588 (37%) | **<0.001** |
| Beta blocker | 12 (22%) | | 102 (6%) | **<0.001** |
| Diuretics | 1 (2%) | | 21 (1%) | 0.750 |
| ACE-inhibitor | 3 (6%) | | 16 (1%) | **0.002** |
| Anticoagulation | 32 (58%) | | 459 (29%) | **<0.001** |

ACE, angiotensin converting enzyme; LVF, left ventricular dysfunction; NYHA, New York Heart Association; VHD, valvular heart disease

| **Table S10** Cardiovascular outcome in valvular heart disease patients | | | | |  |
| --- | --- | --- | --- | --- | --- |
|  | **Total VHD cohort**  N = 1648 | **Impaired LVF**  N = 55 | **Normal**  **LVF**  N = 1593 | **P-value** |  |
| MACE | 325 (20%) | 21 (38%) | 204 (19%) | **<0.001** | |
| Maternal mortality | 17 (1%) | 1 (1.8%) | 16 (1%) | 0.560 | |
| Hospital admission for cardiac reasons | 299 (18%) | 14 (26%) | 285 (18%) | 0.150 | |
| Heart failure episode during pregnancy | 263 (16%) | 17 (31%) | 246 (15%) | **0.002** | |
| Heart failure episode post-partum | 32 (2%) | 2 (4%) | 30 (2%) | 0.350 | |
| Ventricular tachyarrhythmia | 14 (1%) | 0 (0%) | 14 (1%) | 0.490 | |
| Atrial fibrillation or flutter | 48 (3%) | 7 (13%) | 41 (3%) | **<0.001** | |
| Endocarditis | 12 (1%) | 0 (0%) | 12 (1%) | 0.520 | |
| Aortic dissection | 0 (0%) | 0 (0%) | 0 (0%) | - | |
| Ischemic coronary event | 1 (0.1%) | 0 (0%) | 1 (0.1%) | 0.850 | |
| Other thrombo-embolic events | 36 (2%) | 1 (2%) | 35 (2%) | 0.850 | |

P-values were calculated between the group with impaired LV function and normal LV function

LVF, left ventricular dysfunction; VHD, valvular heart disease

1.
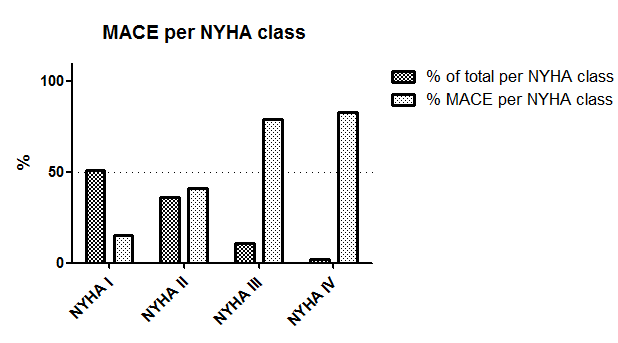
**Supplementary Figures**

Figure S1 Percentage MACE per NYHA class in women with diminished LVF
